# Selective CW Laser Synthesis of MoS_2_ and Mixture of MoS_2_ and MoO_2_ from (NH_4_)_2_MoS_4_ Film

**DOI:** 10.3390/mi15020258

**Published:** 2024-02-09

**Authors:** Noah Hurley, Bhojraj Bhandari, Steve Kamau, Roberto Gonzalez Rodriguez, Brian Squires, Anupama B. Kaul, Jingbiao Cui, Yuankun Lin

**Affiliations:** 1 Department of Physics, University of North Texas, Denton, TX 76203, USA; noahhurley@my.unt.edu (N.H.); bhojrajbhandari@my.unt.edu (B.B.); stevekamau@my.unt.edu (S.K.); roberto.gonzalezrodriguez@unt.edu (R.G.R.); brian.squires@unt.edu (B.S.); jingbiao.cui@unt.edu (J.C.); 2 Department of Materials Science and Engineering, University of North Texas, Denton, TX 76203, USA; anupama.kaul@unt.edu; 3 Department of Electrical Engineering, University of North Texas, Denton, TX 76203, USA

**Keywords:** laser material processing, MoS_2_, (NH_4_)_2_MoS_4_, laser synthesis, laser direct writing, laser-directed thermolysis, Raman, photoluminescence, nanopattern, thermal annealing

## Abstract

Very recently, the synthesis of 2D MoS_2_ and WS_2_ through pulsed laser-directed thermolysis can achieve wafer-scale and large-area structures, in ambient conditions. In this paper, we report the synthesis of MoS_2_ and MoS_2_ oxides from (NH_4_)_2_MoS_4_ film using a visible continuous-wave (CW) laser at 532 nm, instead of the infrared pulsed laser for the laser-directed thermolysis. The (NH_4_)_2_MoS_4_ film is prepared by dissolving its crystal powder in DI water, sonicating the solution, and dip-coating onto a glass slide. We observed a laser intensity threshold for the laser synthesis of MoS_2_, however, it occurred in a narrow laser intensity range. Above that range, a mixture of MoS_2_ and MoO_2_ is formed, which can be used for a memristor device, as demonstrated by other research groups. We did not observe a mixture of MoS_2_ and MoO_3_ in the laser thermolysis of (NH_4_)_2_MoS_4_. The laser synthesis of MoS_2_ in a line pattern is also achieved through laser scanning. Due to of the ease of CW beam steering and the fine control of laser intensities, this study can lead toward the CW laser-directed thermolysis of (NH_4_)_2_MoS_4_ film for the fast, non-vacuum, patternable, and wafer-scale synthesis of 2D MoS_2_.

## 1. Introduction

Since the mono-layer graphene was demonstrated through exfoliation in 2004 [1], two-dimensional (2D) materials have been one of the hot research topics in the field of chemistry, physics, and electronics for their applications in future sensors, transistors, memristors, and single-photon emitters [2,3,4,5,6,7,8,9,10,11,12,13]. At the same time, researchers started the synthesis of large-area 2D materials and developed the chemical vapor deposition (CVD) method [14]. Currently, the 2D materials from the family of transition-metal dichalcogenide [3,15,16,17,18] are targeted for developing next-generation electronic materials due to their unique properties such as direct or indirect band gap modulation and quantum confinement. Large-area thin films of transition-metal dichalcogenide can be fabricated via CVD [17,19]. However, the CVD-based synthesis is limited by the requirement of long processing time and high temperatures (≈1000 °C).

In the micro/nano-fabrication field, the solution-based material process is economically favored because it can start with a spin-coating of wafer-scale films, is compatible with existing CMOS micro/nano-fabrication processes, can be integrated onto various functional substrates, and is especially scalable at low cost and with rapid synthesis [19]. High-quality MoS_2_ or WS_2_ films have been achieved using a single-source precursor such as ammonium tetrathiomolybdate (ATM) (NH_4_)_2_MoS_4_ or (NH_4_)_2_WS_4_, respectively, through thermolysis for electronic devices applications [20,21]. The thermogravimetric analyzer (TGA) curve for (NH_4_)_2_MoS_4_ has shown a possible conversion pathway of (NH_4_)_2_MoS_4_ to MoS_2_ [22]. In the temperature range of 150–330 °C, there is an initial conversion of (NH_4_)_2_MoS_4_ to MoS_3_, and then decomposition of the intermediate MoS_3_ to MoS_2_ in the temperature range of 380–500 °C [22]. Although (NH_4_)_2_MoS_4_ can be dissolved in DI water, the formed film quality is poor. Through the mixing of (NH_4_)_2_MoS_4_ precursor with a polymer matrix polyethyleneimine (PEI), a high quality (NH_4_)_2_MoS_4_ can be prepared, leading to MoS_2_ on a 6-inch SiO_2_/Si wafer [23,24]. Very recently, a laser synthesis of MoS_2_ and WS_2_ through the pulsed laser (λ = 1.06 μm, pulse duration ~100 ps)-directed thermolysis in ambient conditions has achieved wafer-scale samples and devices [2,23,25].

Laser direct writing cannot only generate 2D and 3D patterns for a top-down process, but also generate atomic layer material such as graphene or nanowires for bottom-up nano-fabrication [26,27,28]. It can also generate desired patterns and atomic layers of MoS_2_ [29]. CW laser has a long coherent length and the holographic interference of CW laser beams can form large area 2D or 3D patterns [30,31,32]. It is exciting to have the laser synthesis of atomic layered materials, such as MoS_2_ using CW laser, and simultaneously in large-area patterns formed through the interference of CW laser beams. Although pure MoS_2_ is useful, the mixture of MoS_2_ and its oxide has been used in memristor devices [13].

In this paper, we are able to realize visible CW laser-directed thermolysis of (NH_4_)_2_MoS_4_ for the formation of MoS_2_, instead of the infrared pulsed laser. We study the laser intensity-dependent thermolysis of (NH_4_)_2_MoS_4_ and the formation of MoS_2_ and oxide. The threshold laser power for the laser synthesis of MoS_2_ is clearly observed although the operation window for the laser synthesis of MoS_2_ is narrow. We also compare the laser synthesis process with thermal annealing.

## 2. Sample Preparation and Measurement Methods

A 2D layered MoS_2_ was mechanically exfoliated from the bulk sample with a thickness larger than 300 nm. Ammonium tetrathiomolybdate (NH_4_)_2_MoS_4_ (99.95%, Thermo Scientific Chemicals, Waltham, MA, USA) was used as it is (i.e., crystals) or prepared as a film. It has a melting point of 300 °C. Preparation of ammonium tetrathiomolybdate film on a glass slide followed the instructions given by the procedure document. A low concentration of ammonium tetrathiomolybdate is desired with less than 0.1 wt%. We mixed 0.1 g of (NH_4_)_2_MoS_4_ crystal powder with DI water [33] and then sonicated it for 20 min. The solution is then hydrothermally heated in water for three hours at 95 °C. In this study, we focus on whether we can achieve the laser synthesis of MoS_2_ from (NH_4_)_2_MoS_4_ and pay attention to the film quality in future research. We drop coated the ATM solution onto glass slides and then dried them at room temperature. Once dry, slides are then exposed to laser light of 532 nm and examined via Raman spectroscopy simultaneously.

Raman and photoluminescence (PL) spectra were measured with a Renishaw inVia Raman Microscope that is equipped with a 532 nm laser with a controllable power by software. A grating of 1800 lines/mm was used for the Raman and PL measurements. A 50× objective lens (numerical aperture NA = 0.5) was used for all measurements. The exposure time was 1 s for Raman and 20 s (also 5 accumulations to improve the signal/noise ratio) for the PL unless specified otherwise. The laser spot size is 20.8 microns when using a 50× objective lens, as measured in another paper [18]. Thus, a laser power of 0.3 mW corresponds to laser intensities of 0.176 kWcm^−2^. Because the setting of laser power is in a simple digital of 0.3, 0.4, 0.5, etc., we leave the plot in the *x*-axis in power (mW) instead of laser intensity.

A line pattern of MoS_2_ is formed through the laser thermolysis of (NH_4_)_2_MoS_4_. For a certain film sample and exposure range, we initially performed Raman measurement with increasing laser power and discovered a laser power threshold for the formation of MoS_2_. Using Raman mapping for a line (a grid of 1 × 50, for example) and a laser power above the laser power threshold, the laser scanned through the (NH_4_)_2_MoS_4_ film at a rate of 1 micron per second. Then, we lowered the laser power below the laser power threshold and performed Raman mapping with a grid covering 6 × 40 micron^2^. The line pattern of MoS_2_ was coated with 3 nm gold and Scanning Electron Microscope (SEM) was measured with COXEM CX-200Plus, Daejeon, Korea.

For the purpose of checking the feasibility of (NH_4_)_2_MoS_4_ becoming MoS_2_, we also carried out the thermal annealing of (NH_4_)_2_MoS_4_ crystal and its film. The thermal annealing was performed with Across International STF 1200 CVD tube furnaces system (Livingston, NJ, USA). Both (NH_4_)_2_MoS_4_ crystals and films on glass slides were put inside a quartz tube. To avoid overpressure inside the quartz tube, the tube pressure was maintained at around 2.9 psi, which helps to create an oxygen-free environment, preventing oxidation during the annealing process. The quartz tube was passed with nitrogen gas at the flow rate of 199.9 standard cubic centimeters per minute, and the furnace was heated from room temperature to 500 °C in 50 min at the rate of 100 °C per 10 min. When the temperature reached a consistent value of 500 °C, it was maintained constant for 30 min under nitrogen gas flow. Both commercial (NH_4_)_2_MoS_4_ and annealed samples were studied by X-ray Diffractometer (XRD) (Rigaku MiniFlex 600, The Woodlands, TX, USA).

## 3. Results

### 3.1. Conversion of (NH_4_)_2_MoS_4_ to MoS_2_ by Thermal Annealing

**Figure 1:**
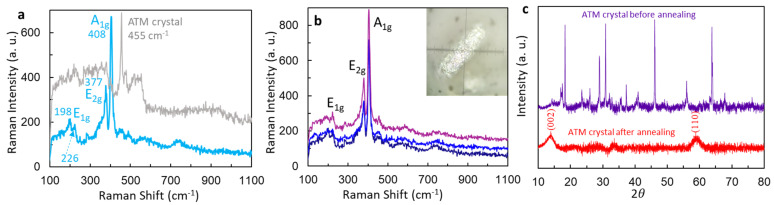
(**a**) Raman spectra at laser power 0.3 mW from ATM crystal (grey line) and from thermally annealed one (blue line) at 500 °C for 30 min. (**b**) Raman spectra of different Raman modes at laser power 0.3 mW from MoS_2_ converted from ATM after annealing, at three different positions. (**c**) Comparison of XRD from ATM crystals before and after thermal annealing at 500 °C for 30 min.

From now on, we use ATM for (NH_4_)_2_MoS_4_. Before we conduct laser thermolysis, we perform the thermal annealing of ATM crystals and their film on a glass slide. Note that while we present the results for an annealed sample here, we actually study the laser thermolysis of ATM earlier. We discovered that laser power below 0.5 mW avoids the laser thermolysis of ATM and oxidation of MoS_2_. Therefore, Raman spectra from the annealed samples were measured under a laser power of 0.3 mW. As shown in Figure 1a, the Raman spectrum has a strong peak at 455 cm^−1^, as expected, for ATM crystal. After annealing, there are three peaks belonging to MoS_2_: the first active Raman peak at 198 cm^−1^ (E_1g_), the second at 377 cm^−1^ (E_2g_), and the third at 408 cm^−1^ (A_1g_) [34]. The intensity for the peak at 198 cm^−1^ (E_1g_) is low. The Raman mode at 377 cm^−1^ (E_2g_) corresponds to out-of-plane vibrations involving only sulfur atoms and 408 cm^−1^ (A_1g_) is due to the opposite vibration of two sulfur atoms with respect to the molybdenum atom [34,35,36]. The peak at 198 cm^−1^ belongs to MoO_2_. By scanning the sample at three different locations in Figure 1b, the Raman peak at 198 cm^−1^ does not show up in the spectrum on the purple line and is very weak in the blue line. It is possible that the laser power of 0.3 mW induced an oxidation, as discussed in more detail later.

The ATM crystal and annealed sample were further studied using X-ray diffraction (XRD). For the XRD from the ATM crystal in Figure 1c, the Bragg reflection peaks at 2θ = 18.3°, 28.9°, 30.8°, and 46.1° corresponds to the crystal faces (hkl) (200), (301), (022), and (413), respectively [22]. There are two Bragg reflection peaks at 2θ = 14 °C and 59 °C, as seen in Figure 1c, corresponding to the MoS_2_ crystal of (002) and (110) [37,38]. It suggests a conversion of ATM into MoS_2_ after the annealing process [37,38].

**Figure 2:**
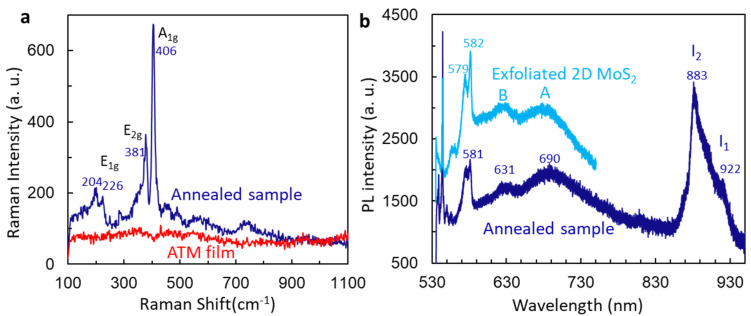
(**a**) Comparison of Raman spectra at laser power 0.3 mW from ATM film and its annealed sample. (**b**) PL measurement for 2D exfoliated MoS_2_ under 10 mW and annealed sample from ATM film at laser power of 0.3 mW.

After sonication and heating at 95 degrees, the ATM film is in amorphous form as seen in the Raman spectrum as shown in Figure 2a. After the ATM film was annealed at a temperature of 500 °C, the Raman spectrum in Figure 2a shows four Raman lines at 204 cm^−1^, 226 cm^−1^, 381 cm^−1^, and 406 cm^−1^ for the ATM films with annellation. Again, the appearance of 204 cm^−1^ shows a trace of MoO_2_ [39]. Figure 2b shows the PL spectra of exfoliated 2D MoS_2_ at 10 mW and annealed ATM film under laser power at 0.3 mW. The PL for exfoliated 2D samples was measured two years ago during the study of stimulated emission [18] and reported for comparison. Both spectra look very similar in the measured wavelength range. The free exciton emission peaks A and B were observed at positions 690 nm (1.79 eV) and 630 nm (1.96 eV), respectively, for both samples [40]. Because we have observed both A and B peaks, and two peaks I_1_ and I_2_ related to indirect bandgap emission at position 992 nm and 883 in Figure 2b, the annealed sample from the ATM film shows multilayer MoS_2_ character.

### 3.2. Laser Thermolysis of (NH_4_)_2_MoS_4_ Crystals

**Figure 3:**
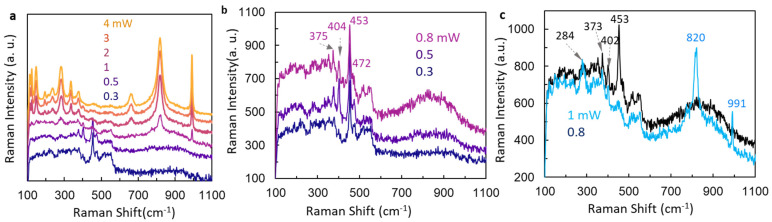
(**a**) Raman spectra from the same location of ATM crystal under laser power 0.3, 0.5, 1, 2, 3, and 4 mW. (**b**) Raman spectra from the same location of ATM crystal under laser power 0.3, 0.5, and 0.8 mW. (**c**) Raman spectra from the same location of ATM crystal under laser power 0.8 mW and 1 mW.

Figure 3 shows overall Raman spectral changes taken at different laser powers ranging from 0.3 mW to 4 mW with the same integration time and at the same sample spot. At the laser power of 0.3 mW, there are two peaks at 453 cm^−1^ and 475 cm^−1^ from ATM crystal. Increasing the laser power to 0.5 mW, peaks were observed at 379 cm^−1^, 404 cm^−1^, 453 cm^−1^, and 475 cm^−1^, as shown in Figure 3a, due to a mixture of ATM and MoS_2_ [38,39,41,42,43,44,45,46]. At 1 mW, the Raman signal due to ATM crystal is gone and the signal of MoS_2_ becomes very weak. However, new peaks at 291 cm^−1^, 824 cm^−1^, and 991 cm^−1^ appear due to the formation of MoO_3_ [38,39,41,42,43,44,45,46]. When the laser power goes above 1 mW, more Raman peaks related to MoO_3_ appear and become stronger.

A more detailed examination of Raman spectra at a smaller laser power step is shown in Figure 3b,c. The Raman signal of MoS_2_ at 379 cm^−1^ and 404 cm^−1^ appears only at 0.5 mW in Figure 3b. At 0.8 mW, the signal/ratio for the Raman peak of MoS_2_ becomes worse. Raman peak at 453 cm^−1^ is always there under these three laser powers. Increasing the laser power from 0.8 mW to 1 mW, the Raman signal at 453 cm^−1^ disappears; thus, the sample has a mixture of MoS_2_ and MoO_3_. From Figure 3, we can see that the sample changes from ATM to a mixture of both ATM and MoS_2_, to a mixture of MoS_2_ and MoO_3_, and then further to MoO_3_ only with increasing laser power. Usually, MoO_2_ can be converted to MoO_3_ above 400 °C [39]. It means that the black-colored ATM crystal can reach a temperature above 400 °C under the CW laser photoexcitation.

### 3.3. Thermal Annealing and Laser Thermal Effects on Exfoliated MoS_2_

**Figure 4:**
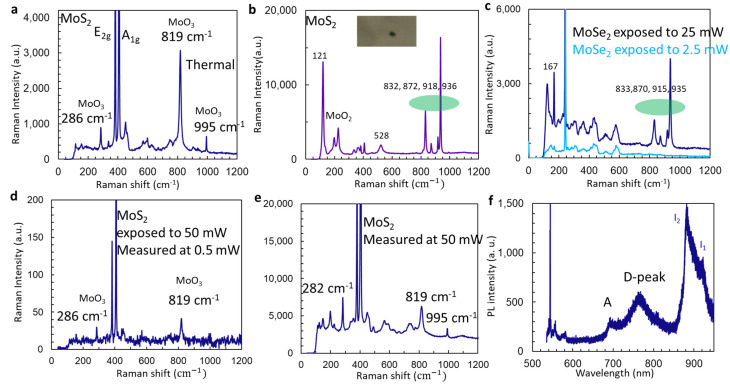
(**a**,**b**) Raman spectra under laser power from non-dark region (**a**) and dark spot ((**b**), image in inset) in exfoliated 2D multilayer MoS_2_ annealed at 420 °C for 30 min in the atmosphere. (**c**) Raman spectra from exfoliated 2D multilayer MoSe_2_ under laser power of 2.5 mW (light-blue line) and 25 mW (black line). (**d**,**e**) Raman spectra from exfoliated 2D multilayer MoS_2_ measured at 0.5 mW after exposed to a laser power of 50 mW for 2 min (**d**) and measured directly under a laser power of 50 mW (**e**). (**f**) PL of exfoliated 2D multilayer MoS_2_ after exposure to a laser power of 50 mW for 2 min.

In order to understand thermal annealing and laser thermal effects on MoS_2_, we study the Raman spectrum of annealed samples of exfoliated MoS_2_ and burned samples of MoSe_2_ under the laser. Multilayer MoS_2_ is very stable up to a temperature of 400 °C at atmospheric conditions. After annealing exfoliated 2D multilayer MoS_2_ on a hotplate at 400 °C for 30 min, Raman modes at 286, 819, and 995 cm^−1^ related to MoO_3_ have been observed in an area without dark spots, together with E_2g_ and A_1g_ modes for MoS_2_, as in Figure 4a. From our observation, oxidation of MoS_2_ occurred first along the defect boundary line and at defect spots. From the defect spot with a dark color in the inset in Figure 4b, the Raman signal of MoO_2_ at 199 and 224 cm^−1^ is observed. Both MoO_2_ and MoO_3_ crystals can have a Raman peak at 528 cm^−1^. However, there are four peaks in the range between 800 and 1000 cm^−1^, at 832, 872, 918, and 936 cm^−1^. These peaks have not been observed in MoO_2_ or MoO_3_. We can assign them to MoO_x_. We have also observed these four Raman peaks at 833, 870, 915, and 935 cm^−1^ in exfoliated 2D multilayer MoSe_2_ excited by a laser power of 25 mW when studying the stimulated emission [47]. It seems these four peaks have nothing to do with “S” or “Se” because we observed these four peaks in both Figure 4b for MoS_2_ and Figure 4c for MoSe_2_.

After we expose the exfoliated 2D multilayer MoS_2_ under a high laser power of 50 mW for 2 min using a 100× objective lens, we are able to observe two weak Raman peaks at 286 and 819 cm^−1^ related to MoO_3_, as shown in Figure 4d. When we directly measured the Raman spectrum under a high laser power of 50 mW using a 100× objective lens and 20 s integration time, we observed three Raman peaks at 286, 819, and 995 cm^−1^ related to MoO_3_ and one Raman peak around 200 cm^−1^ related to MoO_2_, as shown in Figure 4e. The weakness of the Raman signal of MoO_3_ or MoO_2_ might be due to the laser thinning effect [48]. The oxide is formed and becomes vapor. After exposure to high laser power, s-vacancy is formed in MoS_2_ and defect peak emission [18] is seen in Figure 4f in the PL spectrum.

### 3.4. Laser Synthesis of MoS_2_ from (NH_4_)_2_MoS_4_ Film Using CW 532 nm Laser

After we studied and understood the laser thermolysis in ATM crystal, the thermal annealing of ATM crystal and its film, and the annealing effect of exfoliated 2D multilayer MoS_2_, we can report the central piece of this publication: the laser synthesis of MoS_2_.

**Figure 5:**
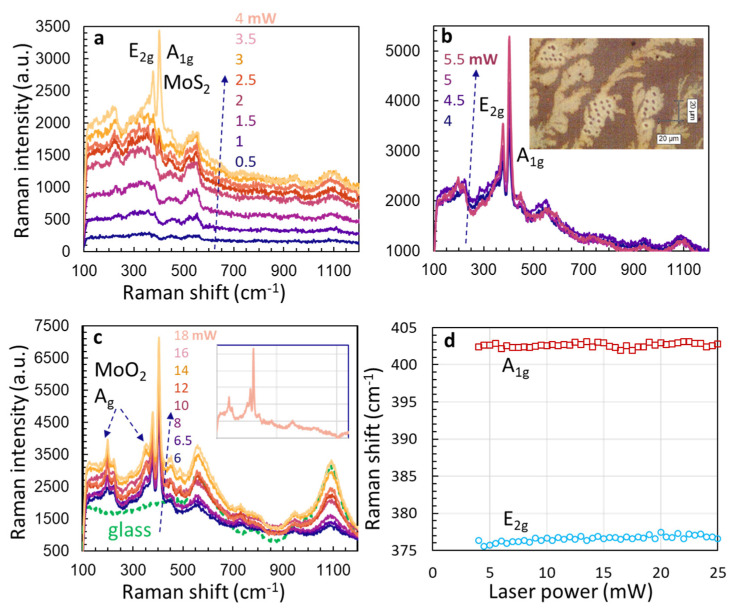
(**a**) Raman spectra from different ATM film locations under a laser power of 0.5, 1, 1.5, 2, 2.5, 3, 3.5, and 4 mW. (**b**) Raman spectra from different ATM locations under a laser power of 4, 4.5, 5, and 5.5 mW. (**c**) Raman spectra from different ATM film locations under a laser power of 6, 6.5, 8, 10, 12, 14, 16, and 18 mW. Inset is the Raman spectrum under a laser power of 18 mW after the spectrum of glass is subtracted. (**d**) Positions of A_1g_ and E_2g_ modes that appear from ATM film under different laser power.

The process started first with a set laser power for the 532 nm laser, then we exposed the sample spot to the 532 nm laser which induced laser thermolysis. This process was repeated by moving to a new location and increased the laser power for laser synthesis of MoS_2_ from ATM film. Figure 5a shows Raman spectra from ATM film under a laser power of 0.5, 1, 1.5, 2, 2.5, 3, 3.5, and 4 mW. Under low excitation laser power, we did not observe the Raman peak 456 cm^−1^ of ATM film. When the laser power goes up to 3.5 mW, several broad bands centered around 242 cm^−1^, 343 cm^−1^, and 460 cm^−1^ are clearly visible. The broad bands are similar to those observed in amorphous ATM that were prepared by heating ATM at 200 °C under N2 [45]. Our ATM preparation under sonication and thermal heating resulted in an amorphous ATM. The broad bands centered at 550 and 1100 cm^−1^ were due to the glass substrate, as discussed later. There is a laser power threshold in the formation of MoS_2_. As seen in Figure 1a, two peaks at 376 and 402 cm^−1^, corresponding to E_2g_ and A_1g_ modes of MoS_2_, appear when the laser power goes from 3.5 to 4 mW. The difference in peak position is 26 cm^−1^, indicating many layers of layered structure are formed in MoS_2_ [23]. Although the laser was excited at different regions as shown in the inset, the laser intensity threshold behavior was confirmed using a new sample.

The formation window of MoS_2_ is narrow, as seen in Figure 5b. When the laser power is increased from 4 to 5.5 mW, a peak at 197 cm^−1^ appears. This peak, together with a peak at 359 cm^−1^, grows stronger when the laser power is increased from 6 to 18 mW in Figure 5c. These two peaks can be assigned to the Ag mode of MoO_2_ [39]. We also measure Raman spectra from the glass substrate (dashed green line) in Figure 1c, confirming the belonging of broad bands centered at 550 and 1100 cm^−1^ to the glass substrate. The inset in Figure 5c shows the Raman spectrum under a laser power of 18 mW after the spectrum of glass is subtracted. After the subtraction, these two broadbands are removed. There is no Raman signal of MoS_2_ and MoO_3_ mixture up to the laser power of 25 mW in the process of laser synthesis of MoS_2_ from ATM film, as we did not observe Raman peaks at 820 and 995 cm^−1^ (see these peaks in Figure 2 and Figure 3) [39]. It also means that the thin ATM film has a reduced laser absorption of laser energy and the temperature of ATM film is not above 400 °C under the CW 532 nm laser photoexcitation.

**Figure 6:**
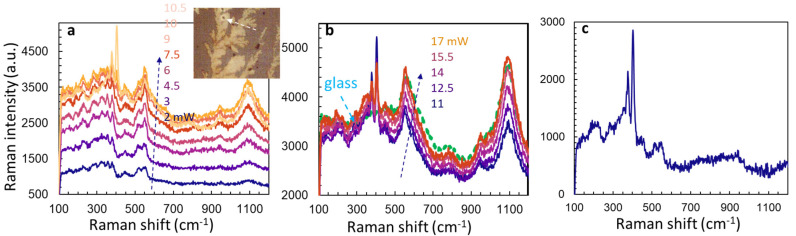
(**a**) Raman spectra from the same ATM film location under a laser power of 2, 3, 4.5, 6, 7.5, 9, 10, and 10.5 mW. (**b**) Raman spectra from the same ATM film location under a laser power of 11, 12.5, 14, 15.5, and 17 mW. (**c**) Raman spectra from the same ATM film location under a laser power of 11 mW after the normalized spectrum of glass is subtracted.

Using a different sample, we then examine the Raman spectra at the same location of ATM film under different laser powers, i.e., measuring the Raman spectrum under one laser power, adjusting the laser power by increasing 0.5 mW for each step then re-measuring the Raman spectrum. When the laser power is increased from 2 to 10 mW, the Raman spectra show broad bands of amorphous ATM, as shown in Figure 6a. Again, a laser power threshold of 10.5 mW for the appearance of E_2g_ and A_1g_ modes of MoS_2_ is observed. As shown in Figure 6b, the peak intensity of E_2g_ and A_1g_ modes of MoS_2_ becomes weaker with the increasing laser power from 11 to 17 mW. However, those broad bands around 550 and 1100 cm^−1^ from the glass substrate become stronger with increasing laser power, indicating that there is a photo-corrosion or laser ablation (laser thinning [48]) of thin film of ATM film and MoS_2_. We linearly normalize the Raman spectrum of glass relative to the Raman intensity of MoS_2_ at a peak position near 1100 cm^−1^ and base position near 780 cm^−1^, then calculate the subtraction as shown in Figure 6c where the broadbands are removed.

### 3.5. Laser Direct Writing of MoS_2_ Line Pattern from (NH_4_)_2_MoS_4_ Film Using CW 532 nm Laser

**Figure 7:**
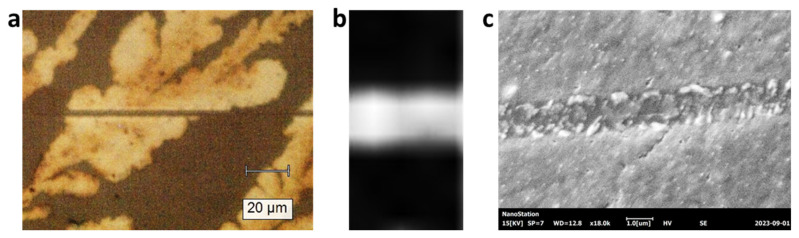
(**a**) Optica microscope image of a line pattern across an ATM film region (white area). (**b**) Raman mapping of the line pattern produced by the laser direct writing using a laser power of 1 mW and integration time of 10 s. (**c**) Scanning electron microscope image of the line pattern.

Through the Raman mapping program in the software, we control the laser beam to scan through in a line. Based on the experimental results in Figure 4, a threshold laser power is 4 mW for the conversion of ATM film to MoS_2_. We set a laser power of 10 mW in order to make sure we can have the laser synthesis of MoS_2_ in every scanning point in the line. The optical microscope image of the scanned line by laser direct writing is shown in Figure 7a, where the line is across a region of ATM film. Then, we lowered the laser power to 1 mW, set the Raman integration to 10 s, and performed Raman mapping in a region covering that line pattern. We selected 402.5 cm^−1^ for the Raman mapping and the results are shown in Figure 7b. Clearly, only a line pattern shows the Raman signal, and other regions are dark (without the MoS_2_ Raman signal). Figure 7c shows a scanning electron microscope (SEM) image of the line pattern by laser direct writing of MoS_2_ from the ATM film. The line pattern is conformed in SEM. The recording resolution is close to 1.5 microns as seen from the width of the line pattern in Figure 7c.

## 4. Discussions

The conversion of ATM to MoS_2_ has been reported by other research groups [20,21]. However, we want to realize the thermolysis of ATM before we carry out the laser thermolysis. The thermal annealing of exfoliated 2D multilayer MoS_2_ helped obtain the Raman feature of MoO_2_ and MoO_3_. The Raman peaks for MoO_3_ have been observed. However, we did not observe the Raman signal of MoO_2_ before the formation of MoO_3_. The origin of four new Raman peaks between 800 and 1000 cm^−1^ is still a puzzle. Because we focus on the laser synthesis of MoS_2_ here, we can study their origin in the future.

Although we have achieved the laser synthesis of MoS_2_ from both ATM crystal and thin film, we see a mixture of MoS_2_ and ATM in the crystal sample. It is reasonable because the crystal volume is very big, while the laser thermolysis occurred in a small volume of ATM crystal. The reported laser power operation window for the laser synthesis of MoS_2_ is very narrow. That operation window can be widened by changing the integration time (i.e., the exposure time for a single spot) or scanning speed if the laser direct writing method is used. On the other hand, the laser wavelength of 532 nm is near the ATM absorption edge [49]; thus, we still have an operation window for the laser thermolysis of ATM using a 50× objective lens. The absorption peak of ATM is around 470 nm [49]. The laser beam can be expanded for the thermolysis of ATM if the laser wavelength is close to 470 nm. We have not studied the film thickness-dependent laser synthesis of MoS_2_. Because the change in the thickness of ATM film will modify the amount of laser energy absorption, it is feasible to achieve laser synthesis of MoS_2_ from ATM film in a wide laser power window.

We did not pay attention to the ATM film quality as we focused on the capability of laser synthesis of MoS_2_ using a CW laser. A uniform micropattern can be obtained using laser radiation in a high-quality film that is needed for electrical and photonic device applications. The high-quality ATM film can be achieved by mixing ATM with a polymer PEI [23,24]. For the device’s capability, an ink printing method [50] can be used. The volume of ink (thus, the thickness of the film) can be precisely controlled and a desired optoelectronic pattern can be printed simultaneously followed by thermal annealing or laser thermolysis. On a high-quality film, laser direct writing [29,51] can be used for a serial fabrication of arbitrary patterns or an interference pattern of CW laser beams [31] can lead to a parallel fabrication of large-area patterns simultaneously in one exposure.

## 5. Conclusions

In summary, we have studied methods for the conversion of (NH_4_)_2_MoS_4_ to MoS_2_ through thermal annealing and laser thermolysis of (NH_4_)_2_MoS_4_ crystals or film, and annealing or laser photoexcitation-induced thermal effect on exfoliated 2D multilayer MoS_2_. For the first time, to the authors’ best knowledge, we have realized the laser synthesis of MoS_2_ from (NH_4_)_2_MoS_4_ crystals film using a CW laser of 532 nm in a certain laser power range. There is a threshold laser power for the conversion of (NH_4_)_2_MoS_4_ film to MoS_2_. Increasing the laser power on the film has resulted in the formation of a mixture of MoS_2_ and MoO_2_ from the ATM film.

## Data Availability

Data will be available upon request.
